# NEK4 suppresses cell proliferation in BT20 triple-negative breast cancer cells by diminishing expression of cell cycle genes, while its depletion mitigates proliferation in other cell lines

**DOI:** 10.3389/fonc.2025.1547899

**Published:** 2025-09-10

**Authors:** Rashmi R. Joshi, E. Gloria Sepulveda, F. Ester Lujan, Jacob M. DeVargas, Amanda K. Ashley

**Affiliations:** Department Chemistry and Biochemistry, New Mexico State University, Las Cruces NM, United States

**Keywords:** NEK4, proliferation, cell cycle, DNA repair, p21

## Abstract

Never In Mitosis Gene A (NIMA)-related kinase 4 (NEK4), a serine/threonine protein kinase, is involved in several cellular processes, including the DNA damage response and mRNA splicing, and we identified it as a potential new drug target in triple-negative breast cancer. We observed via live-cell imaging that multiple NEK4-depleted cell lines proliferated more slowly than control cells, except the BT20 and MCF12A cell lines, in which proliferation was stimulated with NEK4 depletion. We hypothesized that NEK4 regulates genes involved in cell proliferation in BT20 cells. NEK4-depleted BT20 cells were subjected to RNA-seq. Enrichment analysis revealed the upregulation of genes involved in cell cycle, mitosis, and G protein-coupled receptor (GPCR) ligand binding and the downregulation of genes involved in mRNA splicing, consistent with previous reports. We used the STRING database to select a subset of cell cycle genes that were upregulated in NEK4-depleted cells. We assessed cell cycle distribution and found a decrease in the percentage of cells in G0/G1 and an increase in G2/M, suggesting an enhanced proliferative phenotype in BT20 cells. NEK4 depletion did not consistently alter the expression of p21, a putative target of NEK4, in the multiple cell lines examined. We explored the role of NEK4 in DNA repair and observed that NEK4 depletion did not impact basal DNA damage levels but did decrease γH2AX foci following exposure to etoposide. Our data indicate that NEK4 depletion differentially alters cell proliferation in varying cell lines, most likely by altering cell cycle regulation. Understanding its role in cell cycle regulation could be valuable in developing therapeutic strategies and patient stratification, as we and others have identified NEK4 as a potential target for new drug development in triple-negative breast cancer.

## Introduction

Triple-negative breast cancer (TNBC) is a clinical subtype of breast cancer lacking nuclear estrogen receptor (ER) and progesterone receptor and not overexpressing the growth factor receptor HER2. Basal-like TNBC is associated with lower survival (1) and is the most common classification of TNBC at 71% compared to non-basal disease (2). Patients with TNBC suffer from poor prognosis (3), including shorter relapse-free survival, higher risk for recurrent disease (4), and an increased risk of dying as a consequence of tumor relapse compared to other breast cancer types (5). While patients with TNBC face an overall poor prognosis, after 5 years, the risk for recurrence and tumor-related death has diminished to approximately the same level as that of non-TNBC (6). This suggests that TNBC patient survival is highly dependent on preventing recurrence and chemotherapy resistance, and novel therapeutic targets are needed. We identified NEK4 as a potential target in TNBC, however we dually observed that NEK4 knockdown potentiated growth in other cell lines.

NIMA-related kinases (NEKs) are serine/threonine kinases found in eukaryotes, including fungi, *Drosophila*, and mammals. The NIMA kinase in *Aspergillus nidulans* is required for the G2/M transition, where mutant cells remained arrested in the late G2 phase and entered mitosis immediately after NIMA kinase function was restored (7). In yeast, NIMA kinase promotes entry into mitosis, likely by localization with CDC2/cyclin B kinase in the nucleus (8), and is required for mitotic spindle formation, chromatin condensation, and proper cytokinesis (9). Humans express 11 NIMA kinases, with functions distinct from those of their fungal counterparts. All the NEKs contain a conserved N-terminal kinase domain and a variable C-terminal domain (10). Human NEK4 has an N-terminal catalytic domain and a C-terminal regulatory domain with five isoforms expressed (11) (12), and it is a 2021 National Institutes of Health (NIH)-designated, understudied kinase (https://commonfund.nih.gov/idg/understudied-proteins). Presently, no evidence exists suggesting that NEK4 has a role in cell cycle regulation (13), although it likely has a role in cellular microtubule dynamics, as it impacts cilium assembly (14) and differentially sensitizes cells to microtubule toxins (15). NEK4 is critical for cilium assembly, where it interacts with RPGRIP1, RPGRIP1L, and two other NEKs, NEK1 and NEK8 (14). Proper ciliogenesis is critical for the full functionality of the kidneys, reproductive tract, nervous system, and other organs. NEK4 may therefore play a critical role in diseases arising due to cilium dysfunction and ciliopathies, including certain types of cancers (16). Loss of primary cilia is associated with cancers of the kidneys, pancreas, and ovaries and promotes carcinogenesis via dysregulation of cell signaling including the MAPK/ERK, Wnt/β-catenin, and Hedgehog pathways (16).

NEK4 suppression induces cellular resistance to the microtubule stabilizer, taxol, while sensitizing cells to vincristine, which disrupts microtubule formation (15). NEK4 colocalizes with Ku70/80 and DNA-PKcs, critical components of the non-homologous end-joining (NHEJ) DNA double-strand break repair pathway. NEK4 promotes DNA-PKcs recruitment to damage sites and phosphorylation of H2AX (17), suggesting a role in the DNA damage response. An *in silico* protein–protein interactome study revealed the interaction of two isoforms of NEK4 with distinct mRNA splicing factors (12). Experimental evidence using an adenoviral E1A minigene transfected into HEK293 cells showed that these distinct interactions result in different splicing patterns with each of the NEK4 isoforms, implying a role for NEK4 in mRNA splicing (12). NEK4 promotes proliferative arrest in fibroblasts (11) and the epithelial-to-mesenchymal transition in lung cancer cells (12). A decrease in NEK4 expression correlates with advanced stages of colorectal cancer (13), suggesting that NEK4 differentially regulates tumor progression and metastasis in various cancer subtypes. In contrast, in clear cell renal cell carcinoma, high expression promotes patient survival (18). Collectively, NEK4 impacts DNA damage, cellular viability, and post-transcriptional mRNA processing. In addition, we have previously reported that NEK4 knockdown diminishes cell proliferation in a manner independent of p53 in squamous cell carcinoma (19). We and others have suggested that NEK4 may be an attractive target for multiple cancer types (18–24). In addition to its attractiveness as a target for multiple carcinomas, NEK4 has also been identified as a potential drug target in neurological disorders (25–31). Considering the multitude of proposed cellular functions for NEK4, including DNA damage response and cell proliferation, we sought to investigate the transcriptional alterations occurring in NEK4-depleted cells, focusing on triple-negative breast cancer cells, which are known to be deficient in DNA damage repair proteins. Examining gene and pathway alterations in a NEK4-deficient background can reveal novel targets for developing targeted cancer therapeutics.

## Materials and methods

### Cell culture

Cells were purchased from ATCC (Manassas, VA, USA) and grown in recommended media; all assays were conducted in cells that had less than 30 passages. Unless indicated, all cell culture reagents were purchased from Life Technologies (Carlsbad, CA, USA). BT20 and BT549 cells were cultured and maintained in Dublecco's Modified Eagle's Medium (DMEM)/F12 media supplemented with 10% Fetal bovine serum (FBS) and 1% penicillin/streptomycin. MCF7 and HEK293 cells were cultured and maintained in DMEM supplemented with 1% l-glutamine, 1% sodium pyruvate, 1% penicillin/streptomycin, and 10% FBS; MDA-MB231 cells were cultured and maintained in DMEM supplemented with 1% penicillin/streptomycin and 10% FBS. MCF10A and MCF12A cells were maintained in DMEM/F12 media supplemented with 5% horse serum, 1% penicillin/streptomycin, 20 ng/mL of epidermal growth factor, 0.1 µg/mL cholera toxin (Sigma-Aldrich, Saint Louis, MO), 10 µg/mL insulin (Sigma-Aldrich, Saint Louis, MO), and 0.5 μg/mL hydrocortisone (Sigma-Aldrich, Saint Louis, MO). All cell lines were grown and maintained in 5% CO_2_ at 37°C and 100% humidity. NEK4 siRNA (10 nM; Qiagen, Hilden, Germany) was transfected using RNAiMax (Life Technologies) for 24 h unless noted otherwise. For siRNA control, a non-targeting (NT) siRNA was transfected (10 nM, Qiagen AllStars Negative Control). NEK4 depletion was confirmed using qPCR and immunoblotting. In all experiments, both technical and biological replicates were used to ensure robust reproducibility; specific statistical tests are outlined per assay.

### qPCR

The RNeasy Mini Kit (Qiagen) was used for RNA isolation from the cells per the manufacturer’s instructions, with on-column genomic DNA digestion. RNA was quantified and stored until further analysis (32). RNA integrity was confirmed via agarose gel electrophoresis. For cDNA synthesis, 1  μg RNA template was reverse transcribed using the iScript cDNA Synthesis Kit (Bio-Rad, Hercules, CA, USA) per the manufacturer’s instructions. qPCR was performed using iTaq Universal SYBR Green Supermix (Bio-Rad) in the CFX Connect Real-Time PCR Detection System (Bio-Rad) to confirm NEK4 knockdown, and GAPDH was used as the housekeeping gene. Prevalidated primers from the QuantiTect Primer Assay (Qiagen, #QT01008973) system were used for NEK4 qPCR. Primer sequences for GAPDH were as follows: 5′ ACA GTC AGC CGC ATC TTC TT 3′, 5′ ACG ACC AAA TCC GTT GAC TC 3′ (designed against NM_002046.7). All the primers were validated using the cDNA serial dilution method. Forward and reverse primers were used at a final concentration of 0.5 μM with 100 ng cDNA. The qPCR conditions for assessing NEK4 knockdown were as follows: 95°C for 3 min, 40 cycles of 95°C (10 s), 56°C (30  s), followed by a melting curve [65°C–95°C with 0.5°C increments (10  s)]. NEK4 Cq values were normalized to GAPDH, and relative expression was calculated using the 2^−ΔΔCq^ method (33).

### Cell proliferation

Cells were seeded in triplicate in 96-well plates (Costar; Corning, NY, USA) and allowed to attach overnight. Cells were transfected as above and incubated in the IncuCyte S3 Live-Cell Analysis System (Essen BioScience, Ann Arbor, MI, USA). Cell confluence was monitored at 6–8-h intervals for varying timelines. Percent confluence was normalized to the mean confluency recorded at 0 h within each siRNA. Percent confluence of control and NEK4-depleted cells was assessed via non-linear least squares regression analysis, and differences between NT and NEK4 were compared using the extra-sum-of-squares F test on GraphPad Prism 6 (San Diego, CA, USA).

### Immunoblotting

BT20 cells were transfected with siRNA as mentioned above in 60-mm dishes. Protein quantification was carried out using the Pierce BCA Protein Assay (Fisher Scientific, Hampton, NH, USA) per the manufacturer’s instructions. Cell lysates were run on Sodium Dodecyl Sulfate–Polyacrylamide Gel Electrophoresis (SDS–PAGE) gels and transferred to Polyvinylidene difluoride (PVDF) membranes. Membrane-bound proteins were blocked with the appropriate blocking buffer (per antibody recommendation) at room temperature for 1 h. The primary antibody [NEK4 (Bethyl Laboratories, Montgomery, TX, USA), phospho-DNA-PKcs (S2056; Abcam, Cambridge, UK), or DNA-PKcs (Abcam, Cambridge, UK)] was diluted in the blocking solution and incubated overnight at 4°C. Membranes were washed in 1X tris-buffered saline with 0.1% Tween-20 (1X TBS-T) followed by 1-h incubation in the appropriate Horseradish peroxidase (HRP)-conjugated secondary antibodies (Jackson ImmunoResearch, West Grove, PA, USA) diluted in blocking buffer. Membranes were washed using 1X TBS-T, and Clarity Western ECL Substrate (Bio-Rad) was applied per the manufacturer’s instructions. The ChemiDoc MP Imaging System (Bio-Rad) was used for assessing chemiluminescence. When measuring cellular apoptosis, the following primary antibodies were used: cleaved Caspase 9 and cleaved Caspase 3 (Cell Signaling Technologies, Danvers, MA, USA). Immunoblotting was carried out as described above.

### Rhodamine phalloidin staining

BT20 cells were plated on a four-well chamber slide. Cells were treated with NT or NEK4 siRNA for 24 h, then fixed in 2% methanol-free formaldehyde for 10 min at room temperature, washed with Phosphate-buffered saline (PBS), and permeabilized with 0.1% Triton X-100 in PBS. Fixed cells were pre-incubated with 1% bovine serum albumin (BSA)/PBS for 20 min. Rhodamine phalloidin staining was executed by applying a 10-µL methanolic stock solution of rhodamine phalloidin into 200 μL of 1% BSA per well, followed by a 20-min incubation. Cells were mounted with the ProLong^®^ Gold reagent containing 4,6-diamidino-2-phenylindole (DAPI) (Thermo Fisher). Images were captured using a Zeiss Axio Imager Z1 motorized microscope equipped with an XCite LED epifluorescence light source, the Apotome 2 structured illumination module, and an Axiocam 506 CCD camera. Statistical cell size analysis was conducted via a t-test.

### Cell cycle distribution

BT20 cells were transfected with either NT or NEK4 siRNA for 120 h as above. Cells were harvested, washed, fixed in ice-cold 70% ethanol, and stored at −20°C. Cells were stained with a staining solution containing 200 µg/mL propidium iodide and 200 µg/mL DNase-free RNase for 20 min. Cells were filtered on a 0.22-µm filter before being analyzed on a BD Accuri C6 flow cytometer (BD Biosciences, San Jose, CA, USA). A minimum of 10,000 events were recorded for each sample, and the data were analyzed using the FCS Express software. Cells were first gated using side scatter versus forward scatter area and then gated using forward scatter height versus forward scatter area to exclude doublets. Cell distribution is presented as a percentage of recorded events.

### RNA sequencing and pathway analysis

RNA from BT20 cells, transfected with NT or NEK4 siRNA, was isolated and quantified as above. RNA was sequenced using Illumina HiSeq 2000 at the National Center for Genome Resources (NCGR) in Santa Fe, NM. The samples were sequenced using paired-end reads with a read length of 150 base pairs and a read depth of 20 million reads per sample. Quality control of sample reads was performed using the FastQC tool. Raw reads from sequencing were processed to remove adapter and primer sequences. Processed sequences were aligned to the Human reference genome using HISAT2. Read counts were generated using the featureCounts software. Differential gene expression was assessed using the DeSEQ2 tool. Significantly differentially expressed genes, i.e., genes with an False discovery rate (FDR) < 0.05 and fold change of >0 for upregulated genes and <0 for downregulated genes, were included for pathway enrichment analysis using Broad Institute’s Gene Set Enrichment Analysis (GSEA) software (http://www.broadinstitute.org/gsea/). Genes were pre-ranked based on the Wald statistic, which is represented by log2 fold change/standard error of log2 fold change. Change in gene expression was considered significant when the Padj value was <0.05. Log2 fold change of >0 was noted as upregulation, while <0 was noted as downregulation (34). Genes were interrogated against the Reactome database provided in the GSEA tool. Leading edge analysis was carried out on significantly enriched pathways (FDR < 0.05) to reveal a subset of genes contributing the most toward the enrichment score for each of the enriched pathways. The STRING database was used to prioritize genes with known interactors that promote cell cycle.

### Immunofluorescence

Cells were cultured on four-well chamber slides overnight and transfected with NT or NEK4 siRNA for 24 h. Cells were treated with Dimethyl sulfoxide (DMSO) or etoposide (30 µM) for 2 h, followed by fixing with 2% paraformaldehyde and permeabilization in 0.1% Triton X-100. Cells were washed and blocked in 1% bovine serum albumin for 30 min. The primary antibody recognizing p-S139-H2AX (γH2AX; Abcam, Boston, MA, USA) was applied in the blocking solution for 1 h. Cells were washed, and the Alexa Fluor 488-conjugated secondary antibody (Invitrogen, Waltham, MA, USA) diluted in the blocking solution was applied for 1 h. Cells were washed, and slides were mounted using ProLong™ Gold Antifade Mountant containing DAPI (Fisher Scientific, Hampton, NH, USA). A minimum of 10 images per sample were captured using a Zeiss Axio Imager microscope. γH2AX intensity was quantified using the ImageJ software. The mean intensity signal of γH2AX was normalized to the DAPI-stained area per image.

## Results

### In most cell lines, NEK4-depleted cells proliferate more slowly than control cells

**Figure 1:**
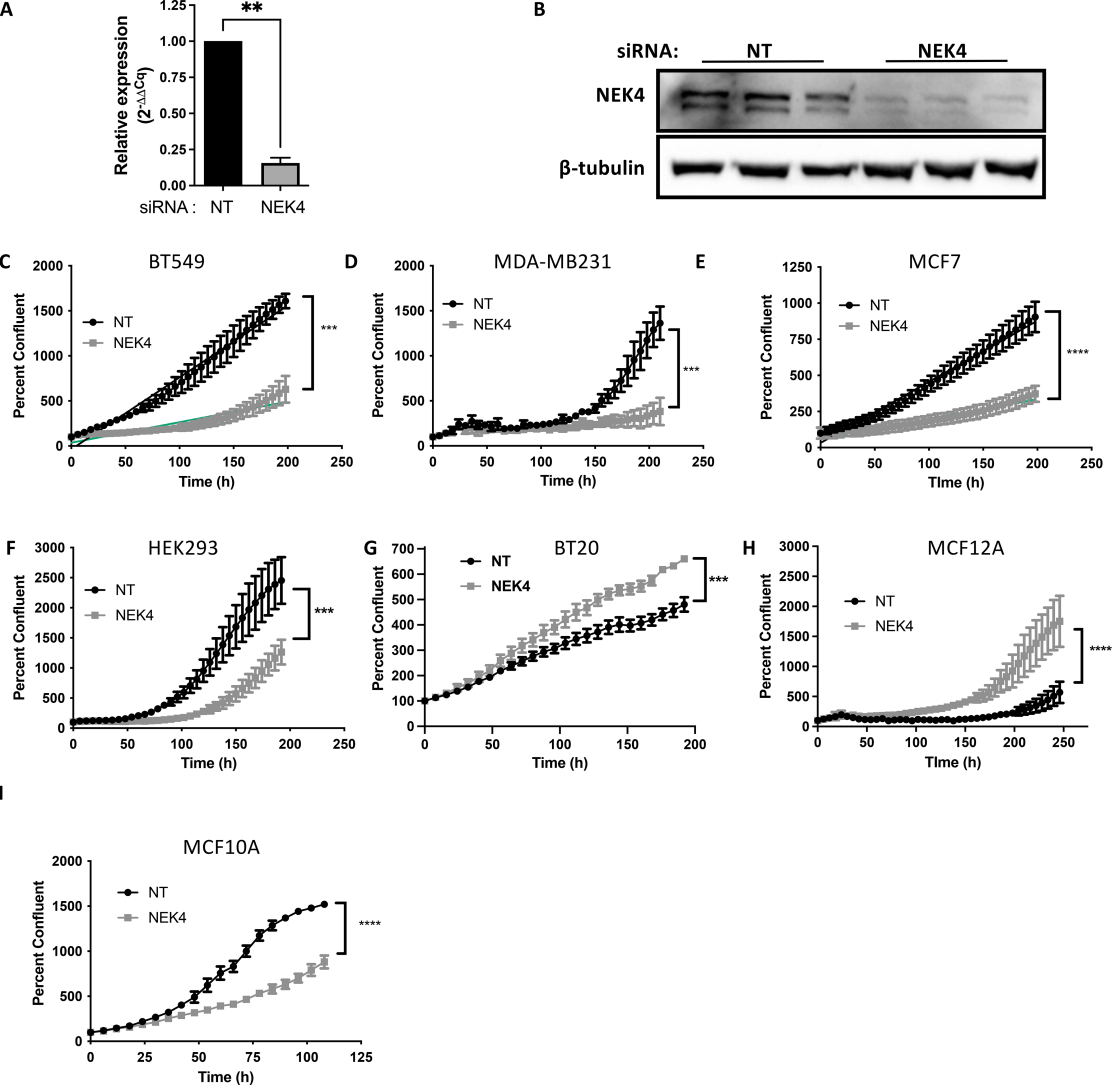
NEK4 knockdown differentially regulates cellular proliferation. BT20 cells were transfected with a siRNA targeting either NEK4 or a scrambled control (NT) for 24 or 48 h NEK4 depletion was confirmed using qPCR **(A)** or Western blotting **(B)** (***p < 0.001, n = 3). NEK4-depleted or control cells were assessed for proliferation using live cell imaging **(C–I)** immediately following transfection. Cell confluence was monitored at 6–8-h intervals for varying timelines. Percent confluence was normalized to the mean confluency recorded at 0 h within each siRNA. Percent confluence of control and NEK4-depleted cells was assessed via non-linear regression, and differences between NT and NEK4 were compared using the extra-sum-of-squares F test (***p < 0.0001, n = 3). **p <0.01; ****p<0.001.

To investigate how NEK4 would impact cell proliferation, we used siRNA to knock down the expression of NEK4 in cells. We identified NEK4 as a possible new drug target in breast cells but were also interested in whether other cell lineages were impacted by NEK4 knockdown, so we employed multiple cell lines: BT20, BT549 and MDA-MB231 (TNBC), MCF7 (ER-positive breast cancer), HEK293 (embryonic kidney cells), and MCF10A and MCF12A (non-tumorigenic triple-negative breast cells). We confirmed knockdown of NEK4 compared to a non-targeting scrambled control (NT) siRNA by qPCR as well as Western blotting in BT20 cells (**Figures 1A, B**) and by Western blotting in MDA-MB231, MCF7, HEK293, BT549, MCF10A, and MCF12A cells (**Supplementary Figures 1A–F**). To assess cell proliferation, we used live-cell imaging where NEK4 or NT siRNA-transfected cells were monitored for confluence over time. Apart from BT20 and MCF12A cells, all cells had reduced confluence over time in NEK4-depleted conditions compared to control cells. In NEK4-depleted BT20 and MCF12A cells, in contrast, confluence increased over time, compared to NT cells (**Figures 1C–I**).

**Figure 2:**
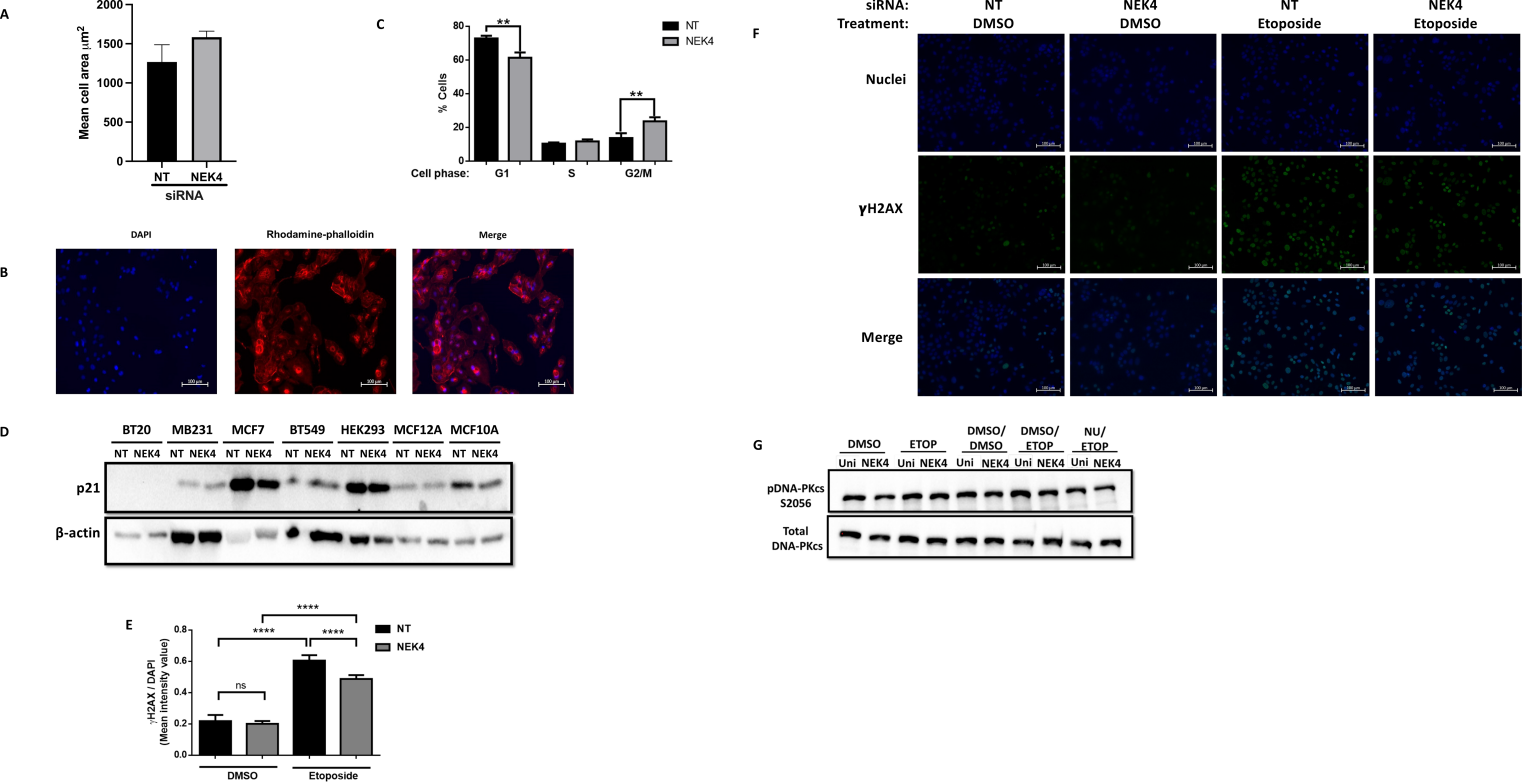
NEK4 depletion does not alter p21 expression but increases percent of BT20 cells in G2/M phase. **(A)** Control and NEK4-depleted cells were stained using rhodamine phalloidin and counterstained with DAPI. **(B)** Representative images of the rhodamine phalloidin-stained cells. **(C)** Cell size was compared using a t-test. Flow cytometric analysis of cell cycle distribution of NEK4-depleted and control BT20 cells reveals a shift in distribution consistent with a pro-growth phenotype (**p < 0.01, n = 3). **(D)** p21 expression was assessed in control and NEK4-depleted cells using Western blotting 120 h post-transfection. β-Actin was used as protein loading control. **(E)** Statistical analysis of NT and NEK4-depleted cells treated for 2 h with 30 µM etoposide or DMSO indicates similar basal levels of γH2AX foci in NEK4-depleted cells. γH2AX foci were quantified using ImageJ software with DAPI-stained area defined as region of interest per image and scored. Mean intensity signal of γH2AX was normalized to the DAPI-stained area per image (****p < 0.0001, n = 5). DNA damage was more pronounced in NT versus NEK4-treated cells. **(F)** Representative image of the γH2AX biomarked cells. **(G)** BT20 cells, transfected with either NEK4 or NT siRNA, were pre-treated with DNA-PKcs inhibitor NU7441 or vehicle (DMSO) for 2 h, followed by a 30-min exposure to etoposide (30 µM) or DMSO and then by protein harvest. Proteins were subjected to SDS–PAGE, transferred onto PVDF, and probed for phospho-DNA-PKcs (S2056) or total DNA-PKcs. Membranes were also probed for the loading control vinculin.

To ascertain whether the altered confluence was due to a change in cell size versus proliferation, we assessed cell size using rhodamine phalloidin staining, confirming that NEK4 depletion did not induce hypertrophy (**Figures 2A, B**). We analyzed the cell cycle distribution of NEK4-depleted cells. We transfected BT20 cells with NT or NEK4 siRNA for 120 h, harvested, fixed, and stained them with propidium iodide. The NEK4-depleted cells increased the percentage of cells in the G2/M phase compared to NT cells with a concomitant decrease in the percentage of cells in the G1 phase; the percentage of cells in the S phase remained similar to that of NEK4-depleted and NT cells (**Figure 2C**). Overall, in most NEK4-depleted cells, proliferation is diminished with NEK4 depletion, but in the BT20 cells, the cells grew more quickly, spending less time in G1 compared to controls.

### Changes in proliferation are independent of p21 expression

NEK4 depletion decreases the expression of the CDK inhibitor p21 in human fibroblasts (35). p21 regulates cell cycle progression in p53-independent and p53-dependent mechanisms (36). We investigated whether changes in p21 expression were correlated with the cellular response of either enhanced or diminished proliferation in NEK4 knockdowns. We assessed the levels of p21 expression in NEK4-depleted and control cell lines (**Figure 2D**) and found that the levels of p21 did not correlate with phenotypic differences observed between cell lines, suggesting that in these cell lines, NEK4 does not regulate cell cycle progression through a p21-dependent mechanism in the cells listed.

### NEK4-depleted BT20 cells exhibit a diminished response to DNA damage

The observation that NEK4 knockdown increases proliferation may be due to erroneous DNA damage recognition and resultant damage signaling, as we have previously observed in cells lacking DNA-PKcs or expressing a mutant variant (37). We assessed whether NEK4 depletion induces the accumulation of DNA damage because of inefficient DNA damage signaling. We quantified the levels of γH2AX, a histone variant phosphorylated in response to DNA damage, and measured γH2AX following NEK4 knockdown alone or knockdown followed by drug challenge with etoposide, a topoisomerase II inhibitor that induces DNA damage. Control and NEK4-depleted cells did not exhibit a significant difference in basal γH2AX foci after DMSO treatment (**Figures 2E, F**). Diminished γH2AX foci in NEK4-depleted cells were observed compared to control cells following etoposide treatment (**Figures 2E, F**). One potential explanation is that NEK4 facilitates the recruitment of DNA-PKcs, one of the kinases that phosphorylate H2AX, to damage sites, where it promotes DNA damage repair (17). DNA-PKcs is autophosphorylated at Ser 2056 following recruitment to DNA damage sites (38). We therefore investigated the levels of DNA-PKcs phosphorylation at S2056 (phospho-DNA-PKcs S2056) as a surrogate for its recruitment to the damage site. We observed that NEK4-depleted BT20 cells had similar levels of DNA-PKcs autophosphorylation as the control cells, indicating that NEK4 depletion does not hamper DNA-PKcs recruitment to the DNA damage site in BT20 cells (**Figure 2G**). Collectively, these results suggest potentially compromised DNA damage signaling in NEK4-depleted BT20 cells; however, further studies need to be conducted to discern the mechanistic details and further explore proteins in the DNA damage response that may be interacting with NEK4.

### NEK4 depletion induces global transcriptome changes in BT20 cells, altering several critical cellular pathways, including cell cycle

Since NEK4 regulates mRNA processing (12), we sought to investigate genes that are transcriptionally regulated by NEK4. BT20 cells were either subjected to siRNA-mediated knockdown of NEK4 or transfected with a non-targeting scrambled control (NT) for 24 h, followed by treatment with PBS for 24 h. RNA was isolated, quantified, and quality confirmed on an agarose gel, and NEK4 knockdown was confirmed using qPCR. Differential gene expression analysis of Illumina sequencing data revealed that NEK4 depletion alone significantly altered expression in 1,992 genes: expression of 1,040 genes was upregulated, while that of 952 genes was downregulated (**Supplementary Figure 3**).

**Figure 3:**
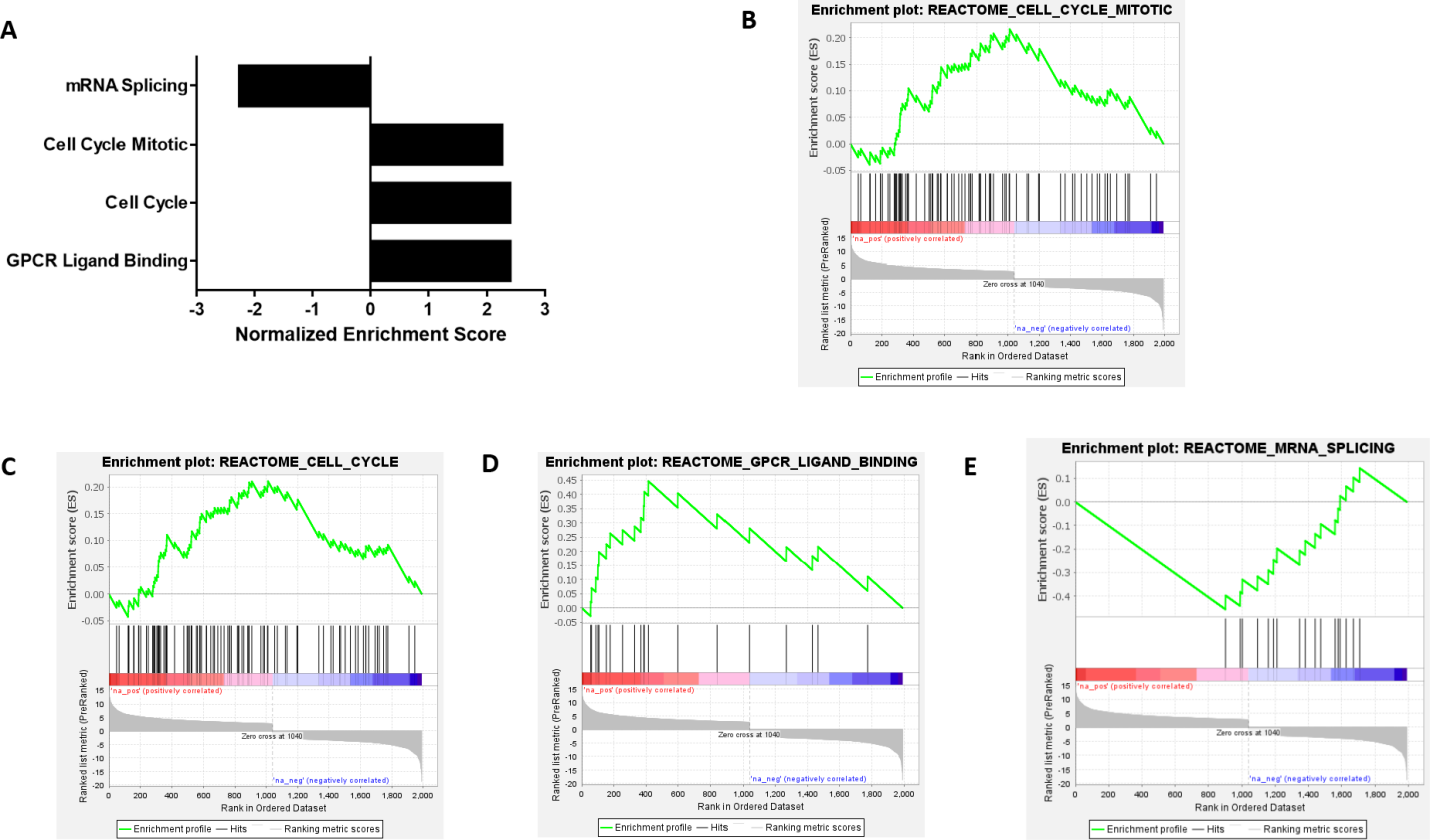
NEK4 depletion upregulates cell cycle genes in BT20 cells. **(A)** Differentially expressed genes were analyzed using Broad Institute’s Gene Set Enrichment Analysis (GSEA). Normalized enrichment score (NES) is suggestive of strength of enrichment. Positive NES indicates upregulation, while negative NES indicates downregulation (FDR < 0.05). **(B–E)** GSEA plots of significantly enriched pathways induced by NEK4 depletion in BT20 cells (FDR < 0.05). Note several key pathways with clinical relevance, including cell cycle and mitotic regulation, as well as GPCR signaling.

To outline what biological pathways were altered due to NEK4 depletion, we utilized the GSEA software from the Broad Institute (**Figure 3A**). We used the pre-ranked GSEA, where significantly altered genes were ranked based on their Wald statistic (log2 fold change/standard error of log2 fold change). We interrogated the REACTOME database, where enriched pathways that had an FDR < 0.05 were considered significant. The significantly enriched pathways were probed via leading edge analysis to enlist genes that contributed the most toward Normalized Enrichment Score (NES). NES signifies the strength of enrichment, with higher (absolute) NES values indicating more robust enrichment (35). Positive NES is indicative of the gene set being overpopulated by upregulated genes, while negative NES indicates that the gene set is populated by downregulated genes, thus being representative of the up- or downregulation of the said pathway. NEK4-depleted cells were positively enriched in processes such as mitotic cell cycle, cell cycle, and G protein-coupled receptor (GPCR) ligand binding (NES = 2.249, 2.387, and 2.393, respectively, FDR < 0.05). The enrichment of cell cycle genes correlates with the observed enhanced proliferative phenotype in NEK4-depleted BT20 cells (**Figure 1G**). mRNA splicing genes were negatively enriched (NES = −2.3, FDR < 0.05) (**Figures 3B–E**).

## Discussion

NEK4 depletion induces a decrease in cell viability in different cancer cell lines as evidenced by our and others’ results (19, 20). We employed siRNA-mediated knockdown of NEK4 in different cancer cell lines and evaluated cellular proliferation via live-cell imaging. Two cell lines increased proliferation following NEK4 depletion for reasons that remain unclear (**Figures 1C–I**) but, as seen in BT20 cells, were associated with alterations in cell cycle gene expression rather than cell size (**Figures 2A–C**). Of note, while the siRNA was efficacious in all cell lines tested, inherent biological variation in the degree of knockdown, as may be expected, was observed but did not correlate with the relevant growth phenotypes. This is in contrast to what we and others have observed in breast cancer (**Figures 1C–E**), human embryonic kidney cells (**Figure 1F**), squamous cell carcinomas (19), and lung cancer and colon cancer tissues. NEK4 depletion promoted tumor necrosis factor-related apoptosis-inducing ligand (TRAIL)-induced apoptosis in TRAIL-resistant lung cancer cells and mouse xenograft models through the downregulation of anti-apoptotic protein survivin (20). NEK4 induces p21-dependent proliferative arrest in human fibroblasts (17); therefore, we measured p21 levels in our differentially responding cell lines with and without NEK4 knockdown. NEK4 depletion was not associated with p21 levels (**Figure 2D**), and our previous studies further rule out differences in p53 (19). Taken together, these data suggest that p21 levels are not consistently altered with NEK4 depletion and that the cell-specific alterations observed in proliferation are independent of p21 and p53 expression (19).

We assessed the transcriptional profile of NEK4-depleted cells to investigate what genes may be responsible for the enhanced proliferation phenotype of BT20 cells. Enrichment analysis of the differentially expressed genes revealed that genes involved in positive regulation of the cell cycle were positively enriched in NEK4 intact cells, further supporting our findings that increased confluence in NT cells over time is due to increased cell proliferation and not cell size (**Figure 3A**). The biological basis for the enhanced proliferation is unclear, but consistent with our previous study, it seems to be independent of p53 status (19), which varies in these cell lines (see **Supplementary Table 1**). In addition, the cell cycle distribution profile of NEK4-depleted BT20 cells revealed an increase in the percentage of cells in the G2/M phase and a resultant decrease in the G1 phase in comparison to the NT cells (**Figure 2C**), indicating increased cell proliferation. Some of the highly upregulated genes are involved in cancer metastasis, including *SPC 25* and *AKT3* (G2M progression), *CCL22* and *CXCL8* (immunoregulatory and inflammation), *KISS1* (metastasis suppression and gonadotrophin-releasing hormone secretion), *WNT7A* (embryogenesis and oncogenesis), and *OXTR* (parturition and lactation). Less abundant expression of genes involved in mRNA splicing following NEK4 depletions was consistent with the literature, suggesting that NEK4 has a role in this pathway (12). Two members of the NEK family of kinases are known to regulate mRNA splicing: NEK4 interacts with several components of mRNA splicing machinery (12), and NEK2 interacts with and phosphorylates mRNA splicing factors (39). In this study, NEK4-depleted cells had less abundant expression of genes involved in mRNA splicing, consistent with the literature stating that NEK4 is involved in mRNA splicing (12). This is the first time that NEK4 has been shown to positively regulate the expression of mRNA splicing genes in addition to its roles in regulating the function of mRNA splicing factors. However, further studies are needed to understand the role of NEK4 in transcriptional regulation of these genes.

In cancer cells, increased proliferation is associated with genomic instability caused by the accumulation of unrepaired DNA damage (40). We measured DNA damage levels using γH2AX foci as markers for damage in NEK4-depleted and control cells. The γH2AX levels were similar in NEK4-depleted cells and vehicle-treated cells (**Figure 2E**). However, decreased phosphorylation of H2AX was observed in NEK4-depleted cells treated with etoposide, a topoisomerase II inhibitor, compared to NT (**Figure 2E**). Other studies have indicated that NEK4 facilitates DNA-PKcs recruitment to the DNA damage site and promotes repair (11). Our data suggest that NEK4-depleted BT20 cells have similar levels of DNA-PKcs phosphorylation as control cells with and without induction of DNA damage following treatment with etoposide (**Figure 2G**). However, we did not directly measure recruitment to the damage site. Others have also suggested that NEK4 has a role in the DNA damage response, positing that it may phosphorylate Ku70 and noting interactions with DNA damage response proteins such as PARP-1 and ERCC6 (12, 17, 41). Further investigations into the role(s) of NEK4 in the DNA damage response are needed.

Our study demonstrates that NEK4 differentially regulates cell proliferation in tumorigenic and non-tumorigenic cell lines in a cell type-specific manner. NEK4 enhances cell death and decreases cell viability and invasiveness in different cancer types, including lung, colon, and head and neck squamous cell carcinomas (19–22). NEK4 upregulation is associated with cell proliferation and metastasis in lung adenocarcinoma (24). Knocking down NEK4 significantly decreases cellular proliferation independent of p53 status (19). NEK4 overexpression also impacts mitochondrial respiration, increasing mitochondrial membrane potential and inducing resistance to mitochondrial DNA damage (42, 43). NEK4 has been suggested as a potential drug target in neurological disorders, including bipolar disorder and migraines (25–31), and control of circadian rhythms (44). We previously identified NEK4 as a target in squamous cell carcinoma (19), and others have found that it may impact colorectal cancer progression (23), whereas high expression of NEK4 promotes patient survival in clear cell renal cell carcinoma (18). We envision future NEK4 inhibitors to be used in combination with DNA-damaging agents to enhance toxicity to cancer cells for improving patient outcomes, but further exploring why some cancers’ growth is potentiated by NEK4 knockdown is critical for proper patient stratification. Incorporating patient-derived xenografts and *in vivo* models will inform whether NEK4 is indeed a potential target for new drug development.

The fact that decreasing NEK4 across multiple cancer subtypes seems to diminish growth independent of p53 status suggests that it may be a promising target in multiple cancer subtypes. The distinct role of NEK4 in regulating cell proliferation and the DNA damage response may intersect with immunotherapeutic strategies targeting PD-L1, particularly in TNBC (45). Utilizing biomimetic carriers such as leukosomes for microRNA-based gene therapy could provide a platform to modulate NEK4 or its downstream pathways, especially as these preferentially hone to areas of inflammation, such as what may be found in a tumor. Furthermore, because leukosomes can evade mononuclear phagocytic system uptake through CD45 (46), they have a biologically long half-life, potentially improving tumor targeting and treatment outcomes. We previously attempted to mimic this concept using mixed silane monolayers in other cancer subtypes (47). Personalized medicine approaches could assist in stratifying TNBC patients based on NEK4 expression or associated microRNA profiles (please see **Supplementary Table 2** for potential miRNA interventions; https://mirdb.org) (48, 49), enabling tailored therapies that target PD-L1 expression (45) or cell cycle vulnerabilities (50) with enhanced precision. Understanding why cell lines differentially respond to changes in the expression of NEK4 milieu has the potential to impact several pathologies and may provide alternative avenues to overcome current treatment limitations, such as in TNBC. Our data present evidence that *NEK4* depletion slows down proliferation in certain cell types while conversely increasing cell cycle progression and cellular division in others. However, further research is needed to understand these opposing phenotypes and their impact on disease progression.

## Data Availability

The original contributions presented in the study are publicly available. This data can be found here: https://doi.org/10.5061/dryad.jm63xsjps.

## Supplementary material

Supplementary Figure 1 Diminished expression of NEK4 protein following siRNA mediated knockdown. Cells were transfected with a siRNA targeting either NEK4 or scrambled control (NT) for 120 h. NEK4 depletion was confirmed using western blot.

Supplementary Figure 2 Patient-derived breast cancer cell lines are sensitive to NEK4 depletion. Cell death is enhanced in breast cancer cell lines derived from TNBC and non-TNBC patients compared to the nontumorigenic human mammary epithelial cell line (HMEC), indicating tumor but not normal cells are sensitive to NEK4 depletion.

Supplementary Figure 3 NEK4 diminishment induces global transcriptomic changes altering critical biological pathways. DESeq2 generated MA plot showing differential gene expression in NEK4 depleted cells compared to control cells (Padj<0.05).

Supplementary Table 1
*TP53* status of cell lines investigated. Assessing the *TP53* mutational landscape of the cell lines interrogated reveals NEK4 knockdown changes cell proliferation independently of *TP53* status.

Supplementary Table 2 miRNAs predicted to interact with NEK4 mRNA. Analysis of miRDB yields miRNAs which might serve to tune the expression of NEK4 expression in triple negative breast cancer.
