# Supplementary figures and images for: NEK4 suppresses cell proliferation in BT20 triple-negative breast cancer cells by diminishing expression of cell cycle genes, while its depletion mitigates proliferation in other cell lines
Source: Front Oncol. 2025 Sep 10;15:1547899. doi: 10.3389/fonc.2025.1547899 (PMC12457296; doi:10.3389/fonc.2025.1547899)

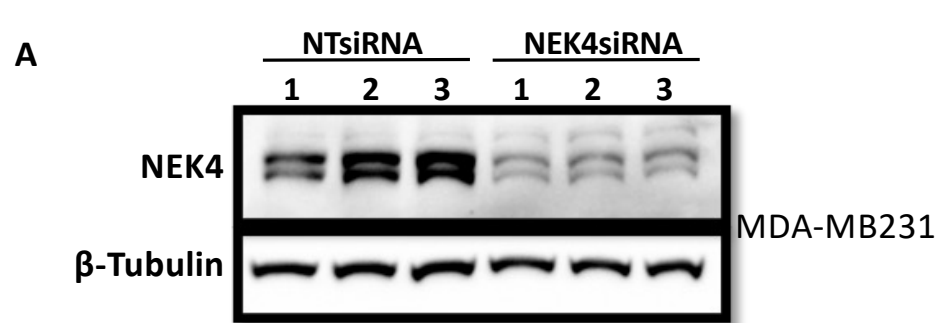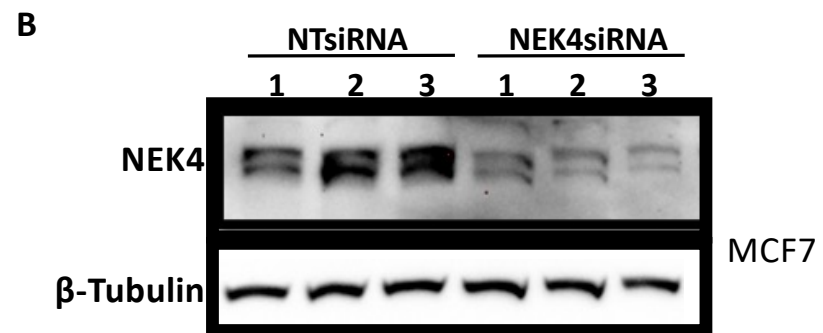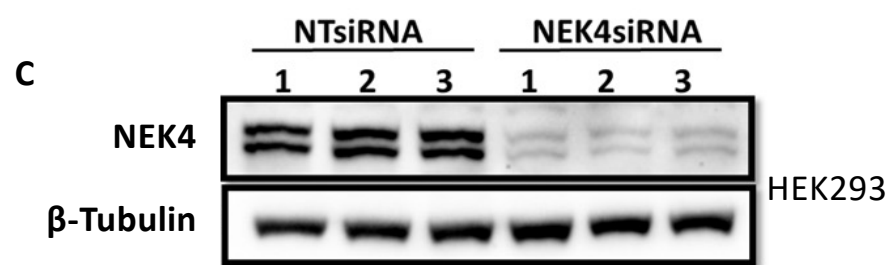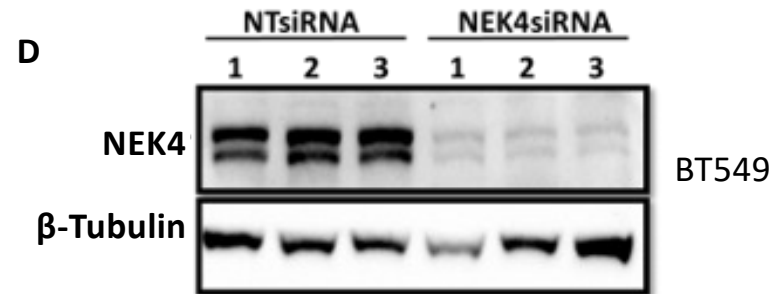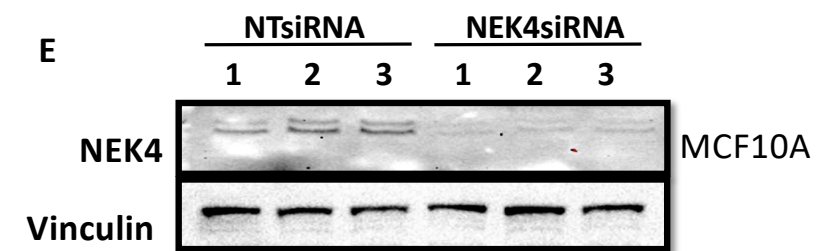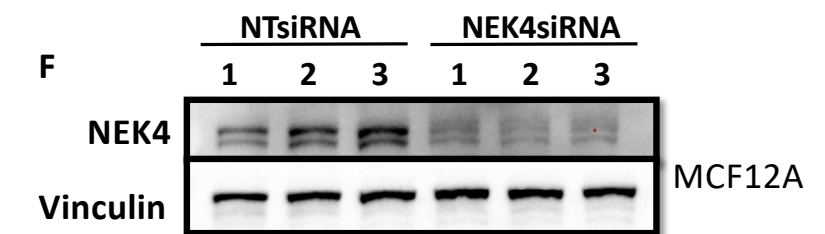

Supplementary Figure 1

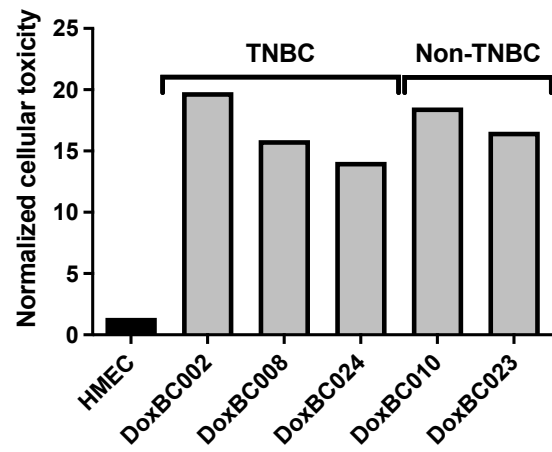

Supplementary Figure 2

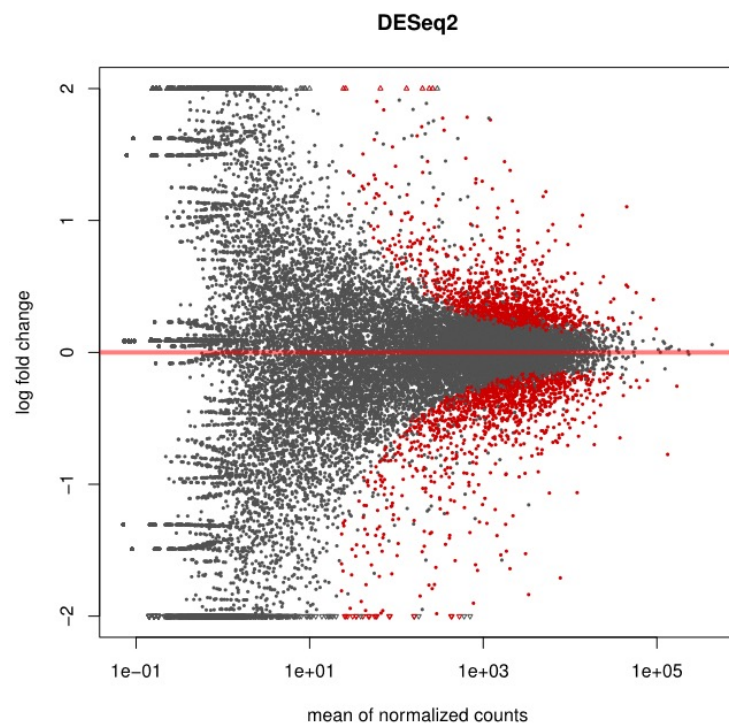

**Supplementary Figure 3**

Supplement: Supplementary Figure 1 — Diminished expression of NEK4 protein following siRNA mediated knockdown. Cells were transfected with a siRNA targeting either NEK4 or scrambled control (NT) for 120 h. NEK4 depletion was confirmed using western blot. [file Image1.pdf]
