# Supplementary material for: NEK4 suppresses cell proliferation in BT20 triple-negative breast cancer cells by diminishing expression of cell cycle genes, while its depletion mitigates proliferation in other cell lines
Source: Front Oncol. 2025 Sep 10;15:1547899. doi: 10.3389/fonc.2025.1547899 (PMC12457296; doi:10.3389/fonc.2025.1547899)
Supplement: Supplementary Table 1 — TP53 status of cell lines investigated. Assessing the TP53 mutational landscape of the cell lines interrogated reveals NEK4 knockdown changes cell proliferation independently of TP53 status. [file Table1.docx]

| **Cell line** | **Type** | **Nucleotide change** | **Residue change** |
| --- | --- | --- | --- |
| BT-20 | Missense | AAG -CAG | Lys (K) - Gln (Q) |
| BT-549 | Missense | AGG-AGC | Arg (R) - Ser (S) |
| MDA-MB-231 | Missense | AGA-AAA | Arg (R) - Lys (K) |
| MCF7 | Wild-type | NA | NA |
| MCF10A | Wild-type | NA | NA |
| MCF12A | Wild-type | NA | NA |
| HEK293 | Wild-type | NA | NA |
|  |  |  |  |
