# Supplementary material for: NEK4 suppresses cell proliferation in BT20 triple-negative breast cancer cells by diminishing expression of cell cycle genes, while its depletion mitigates proliferation in other cell lines
Source: Front Oncol. 2025 Sep 10;15:1547899. doi: 10.3389/fonc.2025.1547899 (PMC12457296; doi:10.3389/fonc.2025.1547899)
Supplement: Supplementary Table 2 — miRNAs predicted to interact with NEK4 mRNA. Analysis of miRDB yields miRNAs which might serve to tune the expression of NEK4 expression in triple negative breast cancer. [file Table2.docx]

| **Target Rank** | **Target Score** | **miRNA Name** |
| --- | --- | --- |
| 1 | 100 | hsa-miR-3163 |
| 2 | 99 | hsa-miR-4282 |
| 3 | 91 | hsa-miR-4328 |
| 4 | 89 | hsa-miR-384 |
| 5 | 87 | hsa-miR-101-3p |
| 6 | 87 | hsa-miR-6868-5p |
| 7 | 85 | hsa-miR-4799-5p |
| 8 | 85 | hsa-miR-4694-3p |
| 9 | 85 | hsa-miR-323a-3p |
| 10 | 84 | hsa-miR-3148 |
| 11 | 84 | hsa-miR-519a-2-5p |
| 12 | 84 | hsa-miR-338-5p |
| 13 | 84 | hsa-miR-520b-5p |
| 14 | 83 | hsa-miR-6165 |
| 15 | 83 | hsa-miR-154-3p |
| 16 | 83 | hsa-miR-545-5p |
| 17 | 83 | hsa-miR-487a-3p |
| 18 | 82 | hsa-miR-3165 |
| 19 | 78 | hsa-miR-3658 |
| 20 | 77 | hsa-miR-1252-3p |
| 21 | 77 | hsa-miR-4311 |
| 22 | 75 | hsa-miR-5093 |
| 23 | 74 | hsa-miR-24-3p |
| 24 | 74 | hsa-miR-3124-3p |
| 25 | 72 | hsa-miR-338-3p |
| 26 | 69 | hsa-miR-138-2-3p |
| 27 | 69 | hsa-miR-516b-5p |
| 28 | 69 | hsa-miR-12129 |
| 29 | 68 | hsa-miR-520d-5p |
| 30 | 68 | hsa-miR-524-5p |
| 31 | 67 | hsa-miR-5586-5p |
| 32 | 67 | hsa-miR-200b-5p |
| 33 | 67 | hsa-miR-627-3p |
| 34 | 67 | hsa-miR-200a-5p |
| 35 | 65 | hsa-miR-302f |
| 36 | 65 | hsa-miR-3688-3p |
| 37 | 65 | hsa-miR-600 |
| 38 | 64 | hsa-miR-3123 |
| 39 | 64 | hsa-miR-3128 |
| 40 | 64 | hsa-miR-580-3p |
| 41 | 63 | hsa-miR-7705 |
| 42 | 62 | hsa-miR-4720-5p |
| 43 | 62 | hsa-miR-4799-3p |
| 44 | 62 | hsa-miR-5588-5p |
| 45 | 61 | hsa-miR-5010-3p |
| 46 | 59 | hsa-miR-4778-5p |
| 47 | 57 | hsa-miR-495-3p |
| 48 | 56 | hsa-miR-656-3p |
| 49 | 56 | hsa-miR-5688 |
| 50 | 54 | hsa-miR-3978 |
| 51 | 54 | hsa-miR-922 |
| 52 | 54 | hsa-miR-3646 |
| 53 | 53 | hsa-miR-5583-5p |
| 54 | 53 | hsa-miR-3140-3p |
| 55 | 53 | hsa-miR-3129-3p |
| 56 | 51 | hsa-miR-549a-3p |
| 57 | 51 | hsa-miR-455-5p |
| 58 | 50 | hsa-miR-1279 |
